# High-Density Genotypes of Inbred Mouse Strains: Improved Power and Precision of Association Mapping

**DOI:** 10.1534/g3.115.020784

**Published:** 2015-07-28

**Authors:** Christoph D. Rau, Brian Parks, Yibin Wang, Eleazar Eskin, Petr Simecek, Gary A. Churchill, Aldons J. Lusis

**Affiliations:** * Departments of Human Genetics, Medicine, Microbiology, Immunology and Molecular Genetics, UCLA, Los Angeles, California 90095-1679; † Department of Anesthesiology, UCLA, Los Angeles, California 90095-1679; ‡ Department of Computer Sciences, UCLA, Los Angeles, California 90095-1679; § The Jackson Laboratory, Bar Harbor, Maine 04609

**Keywords:** mouse, genotyping, mouse diversity array, HMDP

## Abstract

Human genome-wide association studies have identified thousands of loci associated with disease phenotypes. Genome-wide association studies also have become feasible using rodent models and these have some important advantages over human studies, including controlled environment, access to tissues for molecular profiling, reproducible genotypes, and a wide array of techniques for experimental validation. Association mapping with common mouse inbred strains generally requires 100 or more strains to achieve sufficient power and mapping resolution; in contrast, sample sizes for human studies typically are one or more orders of magnitude greater than this. To enable well-powered studies in mice, we have generated high-density genotypes for ∼175 inbred strains of mice using the Mouse Diversity Array. These new data increase marker density by 1.9-fold, have reduced missing data rates, and provide more accurate identification of heterozygous regions compared with previous genotype data. We report the discovery of new loci from previously reported association mapping studies using the new genotype data. The data are freely available for download, and Web-based tools provide easy access for association mapping and viewing of the underlying intensity data for individual loci.

The advent of high-density DNA genotyping has revolutionized the ability of geneticists to identify genes associated with polymorphisms that contribute to common diseases and complex traits. Using genotyping technologies, researchers can now assay hundreds of thousands of single nucleotide polymorphisms (SNPs) in human cohorts in tens of thousands of subjects. To date, more than 6900 loci have been associated with phenotypes ranging from cancer to neurological, cardiovascular, and metabolic disorders in human populations (genome.gov/gwas). Genome-wide association studies (GWAS) often have the precision to identify single candidate genes but in many cases the biological mechanisms that underlie these associations remain uncertain. The process of moving from a locus to a gene to a mechanism is challenging and often requires follow-up studies in model organisms, especially rodents.

Genetic mapping also can be carried out directly in rodent models, and when similar phenotypes are ascertained, it is highly likely that the biological processes that lead to a disease will be shared between humans and mice. Associations of disease phenotypes to polymorphisms in the mouse provide a direct means to identify disease models to support mechanistic studies. Classical approaches to genetic analysis in rodents use low-resolution mapping crosses and correspondingly low-density genotyping is sufficient to achieve the (limited) maximal resolution available from these studies. Recognition that the potential for high-resolution mapping in rodents was not being realized lead to the development of new strategies and resources (Threadgill *et al.* 2002). New resources that have been developed include collections of existing inbred strains of mice (Bennett *et al.* 2010; Ghazalpour *et al.* 2012) as well as the construction of new panels of genetically diverse strains (Collaborative Cross Consortium 2012). In addition, there has been increased interest in the use of outbred rodent populations for genetic mapping studies (Yalcin *et al.* 2010; Svenson *et al.* 2012). Here, we focus on the Hybrid Mouse Diversity Panel (HMDP), which consists of a collection of approximately 175 strains, of which approximately 30 are “classic” inbred strains and 145 are recombinant inbred strains derived from pairs of inbred strains. Generally, about 100 strains are required for sufficient power to map typical complex traits (Bennett *et al.* 2010). The HMDP has been used to examine the genetics of a wide array of phenotypes, including plasma lipids (Bennett *et al.* 2010), bone density (Farber *et al.* 2011), blood cell traits (Davis *et al.* 2013), conditioned fear responses (Park *et al.* 2011), gene-by-diet interactions in obesity (Parks *et al.* 2013), inflammatory responses (Orozco *et al.* 2012), hearing loss (Ohmen *et al.* 2014), diabetes (Parks *et al.* 2015), and heart failure (Rau *et al.* 2015). In many of these studies, genes at the identified loci were validated as causal using engineered mouse models and a number of the loci or genes corresponded to human GWAS results.

Until recently, mapping studies using the HMDP have relied on a set of ∼140,000 SNP loci that were ascertained from multiple sources and merged, including data from the Broad Institute (Kirby *et al.* 2010) and the Welcome Trust Center for Human Genetics (http://mus.well.ox.ac.uk/mouse/INBREDS/). Gaps were filled using imputation to create a uniform set of SNPs for each strain (http://mouse.cs.ucla.edu/mousehapmap/). Here we describe genotyping of ∼650,000 SNP loci for the 175 strains in the HMDP using the Mouse Diversity Array (MDA) (Yang *et al.* 2010; Didion and de Villena 2013). These results complement a previous effort to examine 198 inbred mouse lines using the MDA (Yang *et al.* 2011b); however, all data has been independently generated, and roughly 80% (138) of the strains are novel compared with Yang *et al.* (2011b). The data have been curated to remove poorly performing SNP probes and to correct a handful of errors in strain identification and the sex of genotyped animals. All probes have been remapped by alignment to the most recent release of the reference mouse genome (GRCm38). Updated probe annotations, genotype calls and raw probe intensity are available for download from the Jackson Laboratory (http://churchill.jax.org/mda). In addition, the MDA genotypes now support the online mapping tool (http://mouse.cs.ucla.edu/emmaserver/). We describe the new genotype data and demonstrate that it improves the performance of GWAS using the HMDP.

## Materials and Methods

### Genotyping

Prior Genotypes: genotypes were obtained as previously described (Bennett *et al.* 2010) through the combination of genotypes from the Broad Institute (http://www.broadinstitute.org/mouse/hapmap) and genotypes from the Wellcome Trust Center for Human Genetics. Genotypes of RI strains were imputed from Wellcome Trust genotypes by interpolating alleles at polymorphic SNPs among parental strains.

MDA DNA isolation and hybridization was performed at the Jackson Laboratories as previously described (Yang *et al.* 2010). Genotype calls were obtained using the MouseDivGeno R package (Didion *et al.* 2012)

All analysis of genotypes was performed using the R programming language.

### GWAS

Association mapping was performed as described in Rau *et al.* 2015. We performed the association testing of each SNP using the Efficient Mixed Model Algorithm (Kang *et al.* 2008). This algorithm corrects for population structure among the HMDP using the following model:

$$y=1_{n}m+xb+u+e$$

where m is the mean, b is the allele effect of the SNP, x is the (n × 1) vector of observed genotypes of the SNPs (using additive coding of 0,0.5,1), u is the random effects due to genetic relatedness with $var\left(u\right)=\sigma_{u}^{2}$ and e is the random noise with $var\left(e\right)=\sigma_{e}^{2}I$. K denotes the identity-by-state kinship matrix estimated from all of the SNPs, I denotes the (n × n) identity matrix and 1n is the (n × 1) vector of ones. Both u and e follow normal distributions. $\sigma_{u}^{2}$ and $\sigma_{e}^{2}$ are estimated using restricted maximum likelihood and computed p values using the standard F test to test the null hypothesis b = 0. Thresholds reported in Rau *et al.* 2015 (P < 4.1E-6 suggestive, P < 4.1E-7 significant) were used in this study as well.

### Data availability

Both raw data (CEL files) and genotypes (SQLite database) are available for download and visualization at http://churchill.jax.org/mda. We offer an online MDA browser to explore raw intensity data for SNPs in a region of interest (Supporting Information, Figure S1). This is useful as a diagnostic tool and to help identify other strains (not in the HMDP) that are likely to share causal variants. GWAS results for data reanalyzed from Rau *et al.* 2015 can be found at http://systems.genetics.ucla.edu/ as well as tools for visualization and analysis of these data.

## Results

### The Mouse Diversity Array

The MDA consists of 623,124 SNP probe sets that uniformly cover the nonrepetitive portions of the mouse genome and 916,269 invariant genomic probes that target regions with segmental duplications (Yang *et al.* 2010). SNPs were selected to represent the genetic diversity of the classical inbred strains, which derive primarily from *Mus musculus domesticus* ancestry, as well as sampling the genetic diversity of other mouse species and subspecies including *M.m. musculus*, *M.m. castaneus and M. spretus*. This selection strategy maximizes the discrimination of strains and as such it does not necessarily reflect phylogenic divergence, especially for wild-derived inbred strains.

In total, DNA samples from more than 1900 inbred strains, hybrids, or wild-caught mice have been hybridized at The Jackson Laboratory using the protocol previously described (Yang *et al.* 2010). In this paper we focus attention on SNP genotype calls obtained from the 175 strains that have been used to comprise the HMDP.

### MDA genotypes improve coverage and identify residual heterozygosity

**Figure 1:**
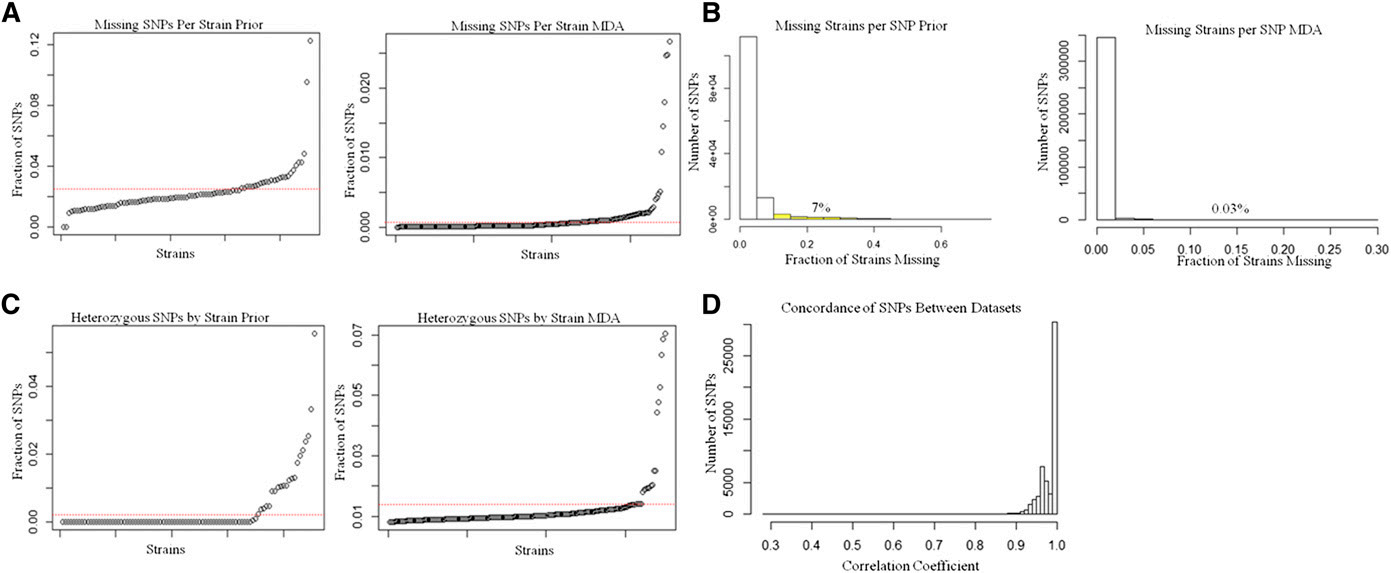
Comparisons of Prior genotypes with Mouse Diversity Array (MDA) genotypes. (A) Fraction of single-nucleotide polymorphisms (SNPs) with missing calls in each strain for Prior (left) and MDA (right) genotypes. The red line indicates the average value. (B) Histogram showing the proportion of missing strains for each SNP for the prior (left) and MDA (right) genotypes. Highlighted in yellow and displayed as a percentage are the numbers of SNPs with more than 10% missing values (7% for prior, 0.03% for MDA). (C) Fraction of heterozygous SNPs within each strain for prior (left) and MDA (right) genotypes. The red line indicates the average value. (D) Histogram of concordance between SNPs found in both genotyping sets.

The previous set of mouse genotypes (Prior) contains ∼140,000 SNPs (Bennett *et al.* 2010), with an average spacing of 20 kb between SNPs. By comparison, the 623,000 SNPs on the MDA have an average spacing of 4.3 kb (Yang *et al.* 2010). We identified ∼550,000 high-quality MDA SNPs and tabulated these by functional classes defined by their location relative to known genomic features (Table 1). As further indications of the quality of the MDA genotypes, we examined the frequency of missing data both within strains and within SNP loci (Figure 1, A and B). Overall, the rate of missing genotype calls was ∼0.1% on the MDA compared with 2.4% on the Prior SNPs (Figure 1A). Only six strains have *more* than 1% missing values in the MDA genotypes, whereas only three strains have *less* than 1% missing values in the Prior genotypes. In the MDA genotypes, we observed 187 SNPs (0.03%) with a missing call rate greater than 10%; in contrast, the Prior genotypes include ∼9800 SNPs (7%) with more than 10% missing values across the 175 HMDP strains (Figure 1B). The increased density of genotyped loci and reduced levels of missing data are important improvements for the identification of GWAS loci as we illustrate below.

**Table 1:** All SNPs present in the Mouse Diversity Array

Total SNPs	∼623,000
Total high-quality SNPs	∼550,000
Intergenic SNPs	∼337,000
Intronic SNPs	∼198,000
Exonic SNPs	∼8,900
3′ or 5′ UTR SNPs	∼5,700

Shown is a listing of the SNPs and their classification on the Mouse Diversity Array. SNP, single-nucleotide polymorphism; UTR, untranslated region.

**Figure 2:**
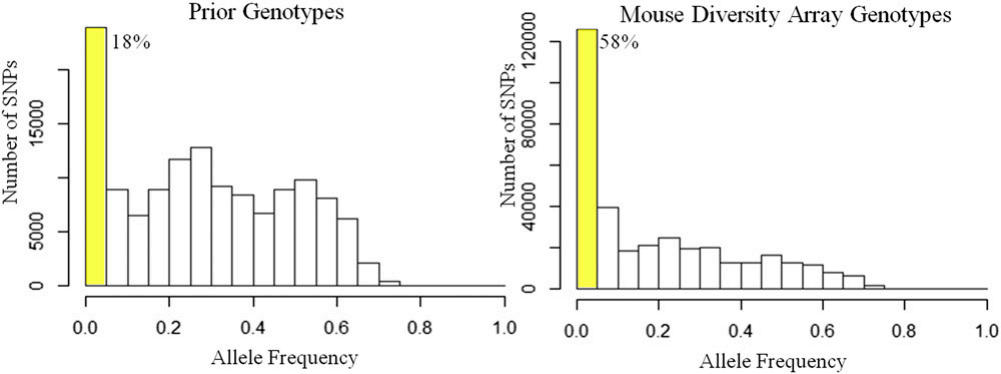
Allele frequencies in genotyping datasets. Histograms of the allele frequency of single-nucleotide polymorphisms (SNPs) in the Prior (left) and Mouse Diversity Array (right) genotypes. Highlighted in yellow and displayed as a percentage are the SNPs whose allele frequencies are too low for genome-wide association studies.

To be useful for mapping, a SNP must be polymorphic in the study population. Furthermore, the minor allele frequency (MAF) should not be too low if used for GWAS to avoid the potential for spurious findings (Figure 2). In our studies we restrict attention to the 42% of high-quality SNPs that have a MAF greater than 5% in the HMDP strains (see Table S1 for a list of strains). In contrast, 82% of SNPs in the Prior genotyping panel have MAF greater than 5%. This difference reflects the selection of SNPs on the MDA, which was designed in part to work with the Collaborative Cross and thus incorporates probes that specifically discriminate among wild-derived strains and are comparatively rare among the common inbred mouse strains (Yang *et al.* 2011a). After eliminating these SNPs as well as SNPs with over 10% missing values, our new genotypes contain 202,473 SNPs (1 SNP per 13.4 kb) that are suitable to genomic analyses. This represents a 1.ninefold increase over the Prior genotypes, which contain 108,565 SNPs (1 SNP per 25 kb) after filtering to remove MAF < 5% and missing values over 10% (Table 2).

**Table 2:** Informative SNPs for performing GWAS in the Hybrid Mouse Diversity panel

	Prior Genotypes	Mouse Diversity Array Genotypes
Total high-quality SNPs	∼140,000	∼550,000
More than 10% missing values	∼9,000	∼200
MAF less than 5%	∼24,000	∼347,300
Final informative SNPs	∼108,500	∼202,500

A comparison of the number of SNPs in both the Prior and MDA genotypes, their reasons for removal and the final number of informative SNPs in each set. SNP, single-nucleotide polymorphism; GWAS, genome-wide association studies; MAF, minor allele frequency.

The HMDP is composed of inbred strains, many of which have been maintained by brother-sister mating for hundreds of generations and are expected to be homozygous throughout their entire genome. However, some of the strains are more recently derived and these may retain regions of residual heterozygosity. The MDA genotypes allow us to gain a better understanding of the heterozygosity remaining in the inbred mouse strains (Figure 1C). The MDA genotype calls are heterozygous at 1.2% of SNPs on average across the HMDP strains. Although the majority of these “H” calls are known to be errors (Didion and de Villena 2013), genomic regions with multiple “H” genotypes in a strain are likely to reflect residual heterozygosity. We note that only a few strains have heterozygous call rates greater than 2%, and all of these are from the more recently derived BXD43-103 panel (Peirce *et al.* 2004).

A common set of ∼71,000 SNPs are represented in both the MDA and Prior genotyping data. Discordant genotypes (Figure 1D) were observed to exceed 10% at only 335 (0.5%) of the common SNP loci. We observed 10 SNP loci with discordance rates greater than 50% between the two data sets. For association analyses, we assumed that the MDA genotypes are correct.

### Improved GWA results

**Figure 3:**
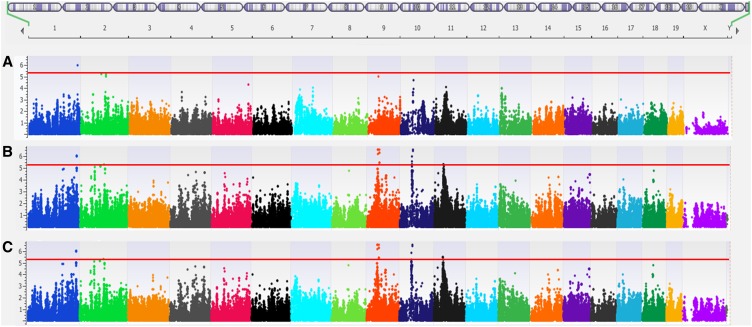
Effects of new single-nucleotide polymorphisms (SNPs) on genome-wide association study results. In both cases, the phenotype being used is total heart weight after isoproterenol treatment. Red line indicates genome-wide significance threshold (4.1E-6). (A) Results using EMMA on the Prior genotypes reveals a single locus on chromosome 1. (B) Results using EMMA on Mouse Diversity Array (MDA) genotypes reveals four additional loci. (C) Results using EMMA on the MDA genotypes using a kinship matrix generated from the Prior genotypes does not demonstrably change the results from B).

To illustrate the improved performance of GWAS using the MDA genotypes, we present a new analysis of previously reported data on the role of catecholamine stimulation on heart weight (Rau *et al.* 2015). We performed GWA analyses of heart weight after catecholamine stimulation using the efficient mixed model algorithm (Kang *et al.* 2008) with both the Prior genotypes (Figure 3A) and the new MDA genotypes (Figure 3B). Using the Prior genotypes, we identified a single significant locus, while using the MDA genotypes, we identified four additional loci at genome-wide significance (*P* < 4.1E-6), as determined previously for the HMDP (Kang *et al.* 2008; Bennett *et al.* 2010). Each of the new loci had achieved a suggestive (*P* < 0.05) level of significance using the Prior genotypes, which provides an indication of the consistency of these findings; however, as only 71,000 SNPs are shared between the two datasets, the specific SNPs making up the peaks in both genotype sets were not entirely identical. Like other mixed-model algorithms (*e.g.*, Lippert *et al.* 2011), EMMA uses a kinship matrix to correct for substructure in the study population. We examined whether changes to the kinship matrix might lead to this result by using the Prior kinship matrix while performing EMMA on the MDA genotypes (Figure 3C, Figure S2, Figure S3, Figure S4, Figure S5, Figure S6, Figure S7, and Figure S8). Although there were some differences in association strengths (Figure S9), the peak SNPs were not affected. When the peak SNP was shared, the SNP had nearly identical genotypes in both sets, which suggests that even small changes to the genotypes can have large effects on the results.

The single significant SNP obtained using the Prior genotypes is located on chromosome 1 ∼25 kb upstream of *Tgfb2*, which has been implicated previously in cardiac morphogenesis and hypertrophy (Lim and Zhu 2006; Azhar *et al.* 2011). The four new loci obtained using MDA genotypes (Table 3) include: *Acvr1*, which has previously been implicated in the regulation of left ventricular heart mass in newborns and congenital defects (Smith *et al.* 2009; Gorący *et al.* 2012); *Drd2*, a gene previously linked to changes in heart rate (Huertas *et al.* 2012) and elevated blood pressure (Rosmond *et al.* 2001); *Pln*, a well-studied gene involved in heart failure (Brittsan *et al.* 1999; Chu and Kranias 2006) and associated by GWAS in human populations with variation in left ventricular internal dimension (Vasan *et al.* 2009); and *Grik2*, a gene that has been associated with heart failure in a human GWAS study (Parsa *et al.* 2011). Although the causal role of these genes remains to be established, their known biology supports a role in determining heart weight after catecholamine stimulation.

**Table 3:** Improved GWAS results due to MDA

Chromosome	Peak SNP rsID	Peak P-value	Distance to Candidate	Candidate Gene	Evidence
Associated in prior genotypes					
1	rs33825648	1.1E-6	55 kb upstream	*Tgfb2*	*Cis*-eQTL, literature
Associated in MDA genotypes					
1	rs33825648	9.8E-7	55 kb upstream	*Tgfb2*	*Cis*-eQTL, literature
2	rs27922490	2.6E-6	2 kb upstream	*Acvr1*	*Cis*-eQTL, literature
9	rs36770705	3.1E-7	Between Exon 4 and 5	*Trim29*	Splicing mutation, literature
9	rs24885538	2.9E-7	Between Exon 2 and 3	*Drd2*	*Cis*-eQTL, literature
10	rs49270079	3.1E-7	737 kb upstream	*Pln*	*Cis*-eQTL, literature
			2.8 mb upstream	*Grik2*	*Cis*-eQTL, literature

Significant loci were observed in both the Prior and MDA genotypes Dashed lines delineate loci from one another. GWAS, genome-wide association studies; MDA, Mouse Diversity Array.

Examination of additional phenotypes reported in Rau *et al.* (2015) (Table 4) shows that the use of the denser MDA arrays led to more significant or suggestive results in each phenotype except for lung weight. Two significant loci reported in Rau *et al.* (2015) were lost in the MDA GWAS: one for liver weight on chromosome 7 over the *Calm3* gene, which is lost entirely, and another for lung weight on chromosome 6 over the *Aqp1* gene, which becomes a suggestive locus. In both cases, the relevant SNPs (rs31334298 for liver, rs30022082 for lung) are present in both genotype sets. In both cases the only difference in genotypes at these SNPs occurred in the C57BL/6J strain, which might explain the large change in association based on the importance of this strain in the HMDP. Ultimately, the MDA genotypes resulted in 24 new suggestive loci and 11 new significant loci when compared to the original results reported in Rau *et al.* (2015).

**Table 4:** MDA leads to many new significant loci compared with results from Rau *et al.* 2015

Phenotype	Prior Genotypes		MDA Genotypes		Lost in MDA Genotypes		Gained in MDA Genotypes	
	Suggestive	Significant	Suggestive	Significant	Suggestive	Significant	Suggestive	Significant
Left ventricle	0	0	2	0	0	0	2	0
Right ventricle	6	3	24	14	2	0	20	11
Left atrium	0	0	1	0	0	0	1	0
Right atrium	1	0	4	0	0	0	3	0
Lung	5	1	4	0	2	1	1	0
Liver	2	1	2	1	1	1	1	1

Suggestive (*P* < 4.1E-6) and significant (*P* < 4.1E-7) thresholds taken from Rau *et al.* (2015). See Table S3 for details about each locus. MDA, Mouse Diversity Array.

## Discussion

Systems-level analyses of complex phenotypes rely on accurate information regarding the underlying genetic variants. Genotypes should be dense enough to ensure that markers are in linkage disequilibrium with most of the potential causal mutations. Equally important in populations with significant levels of population structure, such as admixed human populations or inbred lines of mice, genotypes should be selected to reflect the intrinsic genetic relatedness of the study population. In this study, we examined the effects of obtaining a denser and more accurate set of genotypes in a population (the HMDP), which had previously been analyzed using GWAS (Rau *et al.* 2015). Our new genotypes, obtained using the MDA, increased the number of informative SNPs typed by 87% and improved the genotype quality since large portions of the previous genotypes were imputed.

A previous study (Yang *et al.* 2011b) reported the genotyping of a set of 198 strains using the MDA. Our study complements this study, with 37 strains overlapping with Yang *et al.* 2011b and 138 previously unreported strains. For the 37 strains in common between Yang *et al.* 2011b and our data, we compared the genotypes and observed that, on average, 99.5% of informative SNPs in our data had the same call in Yang *et al.* 2011b, 0.49% were homozygous in either Yang or the present study but heterozygous in the other and 0.01% had a SNP in one data set but not the other (Table S2). These differences are likely the result of either technical error or genetic drift in the inbred lines.

We observed significant improvements over the previously reported GWAS data, returning over double (33 *vs.* 14) the number of suggestive loci in the GWAS study examined here. We explored whether changes to the kinship matrix played a role in this improvement but saw very few changes by switching out one kinship matrix for another. Rather, the new loci appear to be the result of a combination of the addition of new SNPs which, perhaps, are in better linkage disequilibrium with causal mutations as well as corrections to the SNP genotypes, especially in the four core strains (A/J, C3H/HeJ, DBA/2J, and C57BL/6J), which contribute the majority of the power of the panel. Our results suggest that the re-examination of previously analyzed results with a more accurate and denser genotype set may lead to the discovery of novel loci and genes of interest, both in mice as well as in human studies.

## 
